# Effects of time-restricted eating with exercise on body composition in adults: a systematic review and meta-analysis

**DOI:** 10.1038/s41366-024-01704-2

**Published:** 2025-01-10

**Authors:** Harry M. Hays, Pouria Sefidmooye Azar, Minsoo Kang, Grant M. Tinsley, Nadeeja N. Wijayatunga

**Affiliations:** 1 Department of Nutrition and Hospitality Management, University of Mississippi, Oxford, MS USA; 2 Department of Health, Exercise Science and Recreation Management, University of Mississippi, Oxford, MS USA; 3 Department of Kinesiology and Sport Management, Texas Tech University, Lubbock, TX USA

**Keywords:** Risk factors, Signs and symptoms

## Abstract

**Background:**

The effects of time-restricted eating (TRE) with exercise on body composition in adults are not clear.

**Objective:**

This meta-analysis aimed to assess the effects of TRE when followed in combination with various forms of exercise, including aerobic, resistance, and combined aerobic and resistance [concurrent] training on body composition.

**Methods:**

Studies published up to May 2023 were searched in EBSCOhost (MEDLINE, CINAHL, SPORTSDISCUS), PubMed, and SCOPUS databases. Fifteen studies, including 338 participants, that evaluated TRE vs. unrestricted eating in individuals performing exercise were analyzed. A random-effects model was used to calculate the weighted mean effect sizes (ES) with 95% confidence intervals (95% CI’s).

**Results:**

According to the pooled results, TRE had a small but significant reduction of fat mass (FM) kg with an effect size of −0.20 (95% CI = −0.28 to −0.13, *p* < 0.001) and on body fat percent (BF%) with an effect size of −0.23 (95% CI = −0.35 to −0.11, *p* < 0.001). The prediction interval ranged from −0.48 to 0.08 for FM and from −0.64 to 0.18 for BF%, respectively. TRE did not significantly alter fat-free mass (FFM) kg compared to control (*p* = 0.07). Furthermore, age, body mass index (BMI), exercise type, study duration, and energy intake did not have a significant impact on the variation in effect sizes according to the subgroup analyses (*p* > 0.05).

**Conclusion:**

TRE with exercise may reduce fat mass compared to an unrestricted eating window exercise-matched control while preserving FFM. However, more studies are needed.

## Introduction

Intermittent fasting (IF) is a term used to describe dietary interventions in which an individual completes periods of fasting alternating with feeding. Many variations of IF have been implemented in the literature, including alternate-day fasting, 5:2 fasting, Ramadan fasting, and time-restricted eating (TRE). According to past research, intermittent fasting may help to achieve weight loss and reduce cardiometabolic risk [1].

TRE typically involves fasting for 12–20 h per day with a feeding window of 4–12 h [2, 3]. TRE may also mitigate cardiometabolic risks [4] and systemic inflammation [5], and reduce fat mass (FM) [6]. Nevertheless, some studies reported no alterations in body weight or composition compared to control groups [7–9], and others indicated reductions [10, 11] or no change [4, 6, 12] in fat-free mass (FFM) between TRE group and the control.

The combination of diet and exercise is often considered to have augmented effects on body composition as compared to diet alone [13]. Thus, the addition of exercise to a TRE protocol may help minimize losses in FFM and enhance the reductions in FM. It is reasonable to hypothesize that exercise and TRE activate tissue-specific and pathway-specific mechanisms. Exercise may promote mitochondrial biogenesis. Both TRE and exercise may improve glucose metabolism by improving insulin sensitivity, with exercise promoting enhanced glucose uptake in muscles through GLUT-4 translocation [14] and TRE reducing overall systemic insulin levels [15]. Similarly, lipid metabolism is potentially improved by enhanced lipid oxidation by muscle with exercise [16] and an increase in lipolysis via TRE [17]. A meta-analysis assessing the effects of intermittent fasting on body composition among resistance-training individuals reported that intermittent fasting had a significant effect on reducing body mass, FM and body mass index (BMI) relative to the non-intermittent fasting group, without significant differences in FFM [18]. However, this meta-analysis included different types of intermittent fasting protocols and did not study all forms of exercise. Thus, there is a lack of clarity regarding the impact of TRE with various exercise modalities on body composition.

The present systematic review and meta-analysis aimed to assess the effects of TRE on body composition outcomes, including FM, body fat percentage (BF%), and FFM in exercising adults. Pooled data from randomized controlled trials and randomized cross-over studies were analyzed.

## Methods

The Preferred Reporting Items of Systematic Review and Meta-Analysis (PRISMA) statement guidelines were followed for reporting [19] and were registered in PROSPERO (ID number: CRD42022345123).

### Data sources and search strategies

A comprehensive literature search was conducted using five databases, including CINAHL, MEDLINE, SPORTDISCUS, PubMed, and SCOPUS between the 18th of July 2022 and to the 8th of May 2023, and additional searching was performed using the reference lists of previously published articles. Details of the search criteria are in the Supplements. Studies published between October 2016 to February 2023 were included.

### Study selection and eligibility criteria

Randomized controlled trials or randomized crossover studies in healthy adults reporting the effect of TRE with exercise on body composition outcomes, including FM, BF%, and FFM, compared to an exercise-matched control group with an unrestricted eating window were considered. Healthy adults were defined as individuals without cardiometabolic or other chronic health conditions other than overweight/obesity, and not on medications for a chronic health condition. Other inclusion criteria were ≥18 years of age, studies with pre- and post-intervention outcomes, and original research articles published in peer-reviewed journals written in the English language. Short-term studies (<4 weeks) and those without a control group that was following an unrestricted eating window or was exercising were excluded.

**Fig. 1:**
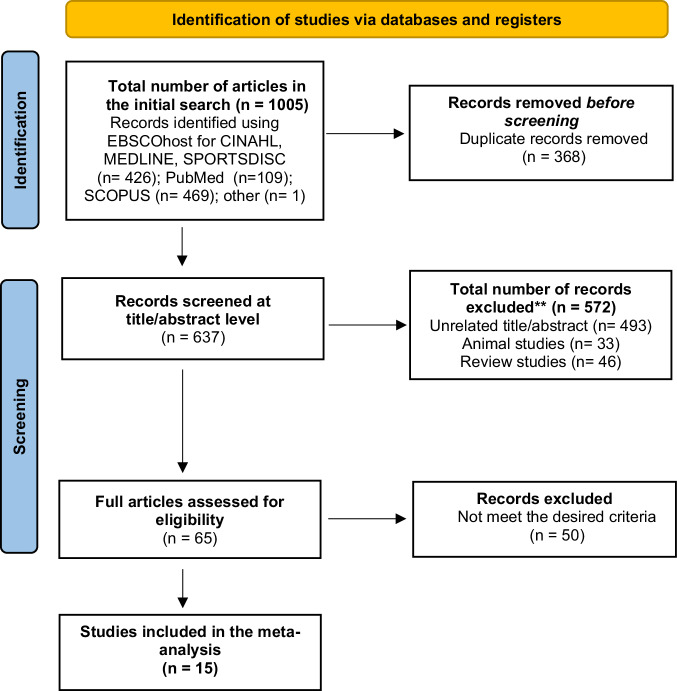
PRISMA flow diagram for the systematic review and meta-analysis. This figure illustrates the different phases of the systematic review and meta-analysis. It includes the number of records identified, screened, assessed for eligibility, and included in the final analysis.

All the articles were exported to Endnote (Version X9.3.3) and duplicates were removed. Articles were screened at the title, abstract, and full-text levels. Two investigators worked separately to perform the search and screening, and any disagreements were resolved by discussion or by a third investigator. The study selection process is summarized in Fig. 1.

### Data extraction

The data extraction and coding were performed by two investigators independently and any disagreements were settled after discussion. The following data were extracted. (1) Study details including authors, publication year, and country; (2) Study sample characteristics including total sample size, sex, baseline BMI, and physical activity level; (3) Study design including study duration, and type of exercise; and (4) Body composition outcomes including pre- and post-intervention BMI, FM, BF%, and FFM. If multiple data collections were present, only the pre-post intervention values were included. Thus, if a study had follow up data after the main study intervention period or if a study had mid-intervention data, such were not included.

### Data synthesis

Three separate meta-analyses were conducted for each of the outcome variables including FM, BF%, and FFM [20]. The mean and standard deviations (SD) for pre- and post-intervention measurements were extracted for intervention and control groups. Next, mean change scores for each outcome variable (post-mean – pre-mean) were calculated. In instances where standard error (SE) was reported instead of SD, SD was calculated by multiplying the SE by the square root of the sample size [20]. The effect size (ES) of each study was calculated using the Campbell Collaboration calculator [21], which computed Cohen’s d (standardized mean difference), 95% confidence interval (CI), and inverse variance weight for each study [22]. Before conducting the meta-analysis, the correlation coefficient (*r*-value) between pre- and post-measurements was determined using the intraclass-correlation coefficient based on previous studies [23–26], because the *r*-value could not be calculated due to a lack of reported SD change scores for all the studies.

Meta-analysis was not conducted for BMI as only 3 out of the 15 studies provided sufficient data to calculate ES for BMI.

### Statistical analysis

A random effects model was used to calculate the weighted mean difference ES and 95% confidence interval (95% CI) for each of the outcomes [20, 21]. As per Cohen’s guidelines, ESs of 0.2 were considered small, 0.5 as medium, and 0.8 as large [22]. The results are reported as the overall ES (weighted mean difference), which are adjusted for sampling error by assigning more weight to studies with larger sample sizes [20].

Cochran’s Q statistic was used to assess the heterogeneity of the overall ES [27]. A Q statistic *p*-value < 0.05 indicates significant heterogeneity among the studies included in the analyses [28]. However, a *p*-value < 0.10 will be considered as significant for the Q statistic in testing heterogeneity in this meta-analysis due to lower power when analyzing fewer studies. Tau-squared statistics (*t*^2^) with a standard deviation of true ES, tau (*t*), and I^2^ statistic were reported with I^2^ statistic representing the percent of the variance in observed effects. The prediction intervals were also reported. The publication bias was assessed using Egger’s regression asymmetry test and by visual inspection of a funnel plot. If publication bias was observed, a trim-and-fill technique [29] was performed.

Moderator analyses (subgroup analysis) were conducted for the moderator variables (BMI, exercise type, intervention duration, and energy intake). ES, 95% CI, and Q-statistics were reported for each moderator analysis. The following moderator variable categories were created. BMI categories were (1) BMI < 25 kg/m^2^ and (2) BMI ≥ 25 kg/m^2^; Exercise categories were (1) aerobic, (2) resistance, and (3) concurrent (both aerobic and resistance); Intervention duration categories were (1) ≥ 8 weeks and (2) < 8 weeks; and Energy intake categories were (1) similar energy intake from pre-to-post between groups, and weight stable (eucaloric) (2) only the TRE group had significantly less ad-libitum energy intake from pre-to-post (TRE hypocaloric), and (3) both groups had significantly less energy intake from pre to post by design, but similar between groups (both hypocaloric). The moderator analysis was conducted using analog to ANOVA analysis. Also, a meta-regression was performed to understand the effects of age (years) on the overall ES.

To convert the ESs to raw scores for better interpretation, the ESs generated by the meta-analysis were multiplied by the pooled SD of FM, BF%, and FFM (kg) in the intervention group from the study with the largest number of subjects. Meta-analyses were conducted using IBM SPSS version 28.0.1.1 (IBM Corporation, Armonk, NY) software program.

### Quality assessment of studies

The risk of bias was assessed independently by two investigators, using the Cochrane Collaboration tool [30]. Any disagreements were resolved by discussion. Briefly, seven items, that were categorized into six domains assessing the risk of bias, were scored as (1) low risk of bias; (2) unclear risk of bias; or (3) high risk of bias. (Table 2).

## Results

### Study characteristics

The initial search resulted in 1005 articles and 368 duplicates were removed. Following initial screening at the title and abstract level, 571 articles were excluded. After a full-text review of the 65 articles, 50 were excluded. Thus, 15 articles were included in the meta-analysis (Fig. 1). The final sample for meta-analysis included 39 effect sizes and 338 total participants from the 15 studies [8, 9, 12, 31–42].

Of the fifteen studies included, seven were conducted in the United States [31, 34, 38–42]. Data of the completers were included in the analysis. All but two studies (which included healthy adults with overweight/obesity) [32, 34] included active, healthy adults with a BMI ≤ 25. Nine of the fifteen studies had all male participants [8, 9, 12, 31, 36, 38–40, 42], and three studies had all female participants [32, 35, 41]. The mean age for all study participants was 28.7 ± 6.52 years. The mean BMI was in the normal to overweight BMI ranges (18.5 kg/m^2^–29.9 kg/m^2^). Of the fifteen studies included, four were aerobic [31, 36, 38, 42], six were resistance [8, 9, 12, 39–41], and five were concurrent training exercise protocols [32–35, 37]. Included in the aerobic training studies were three studies that were conducted in runners [31, 38, 42] and one study was conducted in cyclists [36]. All the resistance training studies included both upper and lower body exercises performed at least three times per week [8, 9, 12, 39–41], and this was generally under supervision. The concurrent training interventions included both resistance training and aerobic training weekly [33, 34, 37] or high-intensity interval training [32, 35]. Study duration was ≥8 weeks except for six studies [8, 9, 32, 36, 39, 42]. In five studies, all participants were in a mild (≤500 kcal) energy restricted state from beginning to end of intervention, [33, 34, 37, 39, 41]. Of those, two of the studies imposed a 20–25% energy restriction for both groups [37, 39] while in one study there was a 250 kcal energy reduction for all groups [41] and other had a 500 kcal reduction in the control group only [33]. Furthermore, TRE led to a mild energy restricted state, pre-to-post intervention in three studies compared to control [32, 35, 40]. Most studies utilized a mid-day TRE protocol, where the feeding window falls between ~12 pm and 9 pm [8, 9, 12, 31, 33, 34, 37–39, 41, 42], three studies utilized a self-selected window [32, 35, 42], and two studies where TRE window was between 10 am to 6 pm [36] and 4 pm to midnight [40]. In all the studies, the exercise sessions were performed during the feeding window. Adherence to TRE was determined through food logs/recalls or adherence questionnaires in all studies and also assessed by weekly conversations with a dietitian in two studies [12, 36]. Table 1 summarizes the characteristics of the 15 studies included in the meta-analysis.

**Table 1:** Characteristics of randomized controlled and cross-over studies included in the meta-analysis (*n* = 15).

Study (year)	Sex	Mean age ± SD (years)	Sample size	Mean BMI ± SD (kg/m^2^)	Intervention length (weeks)	Exercise type	TRE protocol; fasting hours: feeding hours	Energy intake	Body composition outcome (compared to non-TRE)
Brady et al. 2021 [31]	Males only	TRE = 35.9 ± 8.6. C = 39.9 ± 3	17	22.3 ± 3	8	Aerobic	16:8; 12 pm to 8 pm	Eucaloric	^b^FM & FFM ↔
Correia et al. 2021 [8]	Males only	22.4 ± 2.8	12	24.2 ± 2	4^a^	Resistance	16:8; 1 pm to 9 pm	Eucaloric	^c^FM, FFM & BF% ↔
Kotarsky et al. 2021 [34]	3 males and18 females	44 ± 7	21	29.6 ± 2.6	8	Concurrent	16:8; 12 pm to 8 pm	Both hypocaloric	^c^FFM ↔, FM ↓
Moro et al. 2016 [12]	Males only	TRE = 29.9 ± 4.07. C = 28.4 ± 3.48	34	TRE = 26.5 ± 1.2; C = 27.3 ± 1.4	8	Resistance	16:8; 1 pm to 8 pm	Eucaloric	^c^FFM ↔, FM ↓
Moro et al. 2020 [36]	Males only	19.3 ± 0.1	16	TRE = 21.85 ± 1.65; C = 22.47 ± 1.83	4	Aerobic	16:8; 10 am to 6 pm	Eucaloric	^b^FFM ↔, FM & BF% ↓
Stratton et al. 2020 [39]	Males only	TRE = 22.9 ± 3.6. C = 22.5 ± 2.2	26	TRE = 25.9 ± 2.3; C = 26.3 ± 1.7	4	Resistance	16:8; 12 pm to 8 pm	Both hypocaloric	^d^FFM, FM & BF% ↔
Tinsley et al. 2017 [40]	Males only	TRE = 22.9 ± 4.1. C = 22 ± 2.4	18	24.3 ± 2	8	Resistance	20:4; 4 pm to midnight	TRE hypocaloric	^c^FFM, FM & BF% ↔
Tinsley et al. 2019 [41]	Females only	22 ± 2.2	24	TRE = 23.6 ± 2.3; C = 22.3 ± 2.2	8	Resistance	16:8; 12 pm to 8 pm	Both hypocaloric	^d^FFM ↔, FM & BF% ↓
Tovar et al. 2021[42]	Males only	28.7 ± 5.2	15	23.2 ± 1.8	4^a^	Aerobic	16:8; Self-selected	Eucaloric	^c^FFM ↔, FM & BF% ↓
Martinez et al. 2021 [35]	Females only	27 ± 6	14	21 ± 1.7	8^a^	Concurrent	14:10; Self-selected	TRE hypocaloric	FFM ↔, FM & BF% ↓
Ribiero et al. 2021 [37]	4 males and 20 females	TRE = 32 ± 5.5. C = 33 ± 8.7	24	TRE = 30.5 ± 3.5; C = 31 ± 5.6	8	Concurrent	16:8; 12 pm to 8 pm	Both hypocaloric	^b^FFM ↑, FM ↔
Isenmann et al. 2021 [33]	14 males and 21 females	TRE = 27.9 C = 27.4	35	TRE = 26.3; C = 25.7	16	Concurrent	16:8; 12 pm to 8 pm	Both hypocaloric	^b^FFM & FM, ↔
Correia 2023 [9]	Males only	23.7 ± 2.6	18	N/A	4^a^	Resistance	16:8; 1 pm to 9 pm	Eucaloric	^c^FFM & FM, ↔
Haganes 2022 [32]	Females only	36.2 ± 6.2	49	TRE = 31.4 ± 4; C = 32.5 ± 4.5	7	Concurrent	Minimum 10 hr fasting	TRE hypocaloric	^b^FFM ↔, FM, ↓
Richardson 2023 [38]	Males only	28.7 ± 5.2	15	N/A	4^a^	Aerobic	16:8; 12 pm to 8 pm	Eucaloric	^c^FFM ↔, FM & BF% ↓

Abbreviations: *Both*, TRE and control groups; *C* Control, *TRE* time-restricted eating, *FM* Fat mass, *FFM* Fat-free mass, *BF%* Body fat percent, ↓ Decreased, ↔ No difference, ↑ Increase; *Eucaloric,* No difference in energy intake between groups pre to post; and *Hypocaloric,* Energy intake less from pre to post.

^a^Random cross-over design.

^b^Bioelectrical impedance analysis.

^c^Dual-energy x-ray absorptiometry.

^d^4-compartment model.

### Quality of individual studies

Findings for the risk of bias assessment using the Cochrane Collaboration’s tool for assessing risk of bias [30] are reported in Table 2. Of the 15 studies, 13 were classified as having a low risk of bias and 2 as unclear risk of bias for allocation concealment. The blinded participant’s assessment criteria were rated as “high risk” for all studies since blinding of participants is not feasible due to the nature of interventions. Furthermore, 2 of the 15 studies rated as having a high risk of bias for selective reporting, and 12 of 15 were rated as unclear risk of bias for blinding of outcome assessment.

**Table 2:** Quality assessment based on Cochranes Collaboration’s tool risk of bias assessment.

Study name	Random sequence generation	Allocation concealment	Blinding of participants and personnel	Blinding of outcome assessment		Incomplete outcome data	Selective reporting		Other bias	Overall bias
Brady et al. 2021 [31]	Low risk	Low risk	High risk	Low risk	Low risk		Low risk	Low risk		Low risk
Kotarsky et al. 2021 [34]	Low risk	Low risk	High risk	Unclear risk	Low risk		Low risk	Low risk		Low risk
Moro et al. 2016 [12]	Low risk	Low risk	High risk	Low risk	Low risk		Low risk	Low risk		Low risk
Moro et al. 2020 [36]	Low risk	Low risk	High risk	Unclear risk	Low risk		High risk	Low risk		Unclear risk
Isenmann et al. 2021 [33]	Low risk	Low risk	High risk	Unclear risk	Low risk		Low risk	Low risk		Low risk
Tinsley et al. 2019 [41]	Low risk	Low risk	High risk	Low risk	Low risk		Low risk	Low risk		Low risk
Stratton et al. 2020 [39]	Low risk	Unclear risk	High risk	Unclear risk	Low risk		High risk	Low risk		Unclear risk
Tinsley et al. 2017 [40]	Low risk	Unclear risk	High risk	Unclear risk	Low risk		Low risk	Low risk		Low risk
Tovar et al. 2021 [42]	Low risk	Low risk	High risk	Unclear risk	Low risk		Low risk	Low risk		Low risk
Martinez et al. 2021 [35]	Low risk	Low risk	High risk	Unclear risk	Low risk		Low risk	Low risk		Low risk
Correia et al. 2021 [8]	Low risk	Low risk	High risk	Unclear risk	Low risk		Low risk	Low risk		Low risk
Ribeiro et al. 2021 [37]	Low risk	Low risk	High risk	Unclear risk	Low risk		Low risk	Low risk		Low risk
Correia et al. 2023 [9]	Low risk	Low risk	High risk	Unclear risk	Low risk		Low risk	Low risk		Low risk
Haganes et al. 2022 [32]	Low risk	Low risk	High risk	Unclear risk	Low risk		Low risk	Low risk		Low risk
Richardson et al. 2023 [38]	Low risk	Low risk	High risk	Unclear risk		Low risk	Low risk	Low risk		Low risk

### The overall changes in the body composition outcomes

**Fig. 2:**
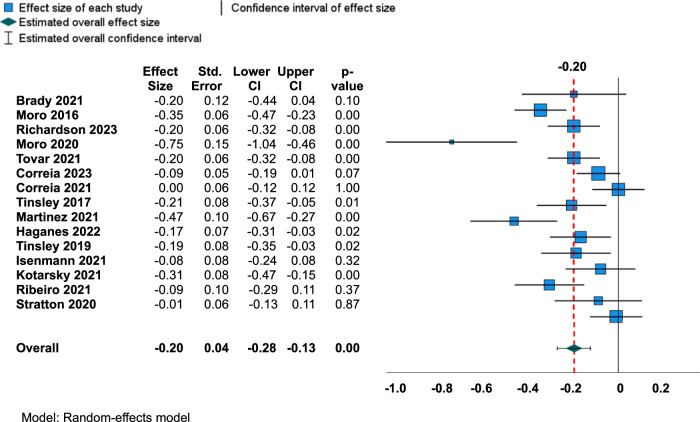
Forest plot for changes in fat mass. Abbreviations: CI Confidence interval.

**Fig. 3:**
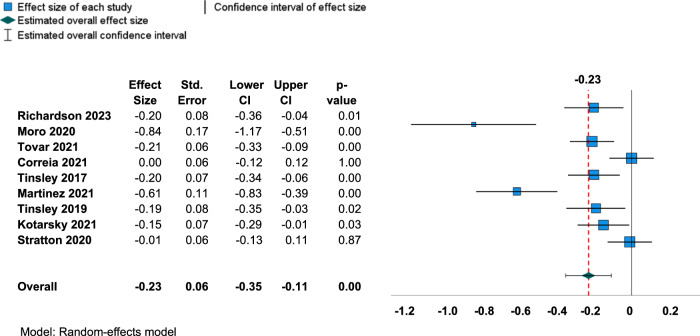
Forest plot for changes in body fat percentage. Abbreviations: CI Confidence interval.

**Fig. 4:**
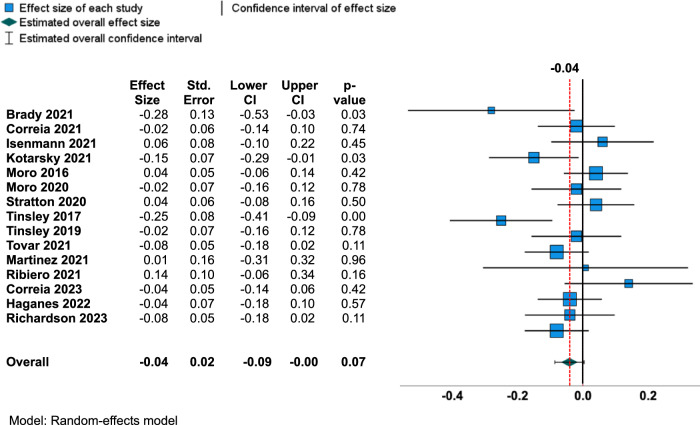
Forest plot for changes in fat-free mass. Abbreviations: CI Confidence interval.

**Fig. 5:**
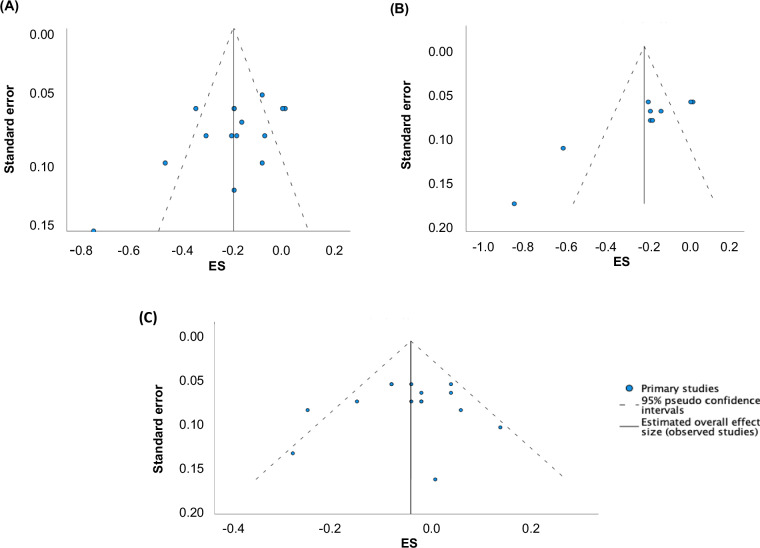
Funnel plots for assessing publication bias. Funnel plots for **A** Fat mass, **B** Body fat percent, and **C** Fat-free mass. Abbreviations: ES Effect size.

The meta-analyses for FM, BF%, and FFM are shown as Forest plots in Figs. 2, 3, and 4, respectively. There was a small but significant mean reduction in FM following TRE with exercise compared to exercise only (ES = −0.20, 95% CI = −0.28 to −0.13, and *p* < 0.001). Since the prediction interval is −0.48 to 0.08, the true ES can be as low as −0.48 in certain populations and as high as 0.08 in others. The weighted mean difference ES observed for FM was multiplied by the pooled SD of FM (kg) of the intervention group with the largest number of subjects from the study that was included in this meta-analysis [32]. Thus, there may be an additional 1.3 kg reduction in FM in the TRE with the exercise group compared to the exercise-only control group. However, high heterogeneity was observed for changes in FM between the studies according to Q statistics (Q = 57.8, df = 14, *p*-value < 0.001), and I^2^ statistic (I^2^ = 75%). The estimate of true between-study variance is τ^2^ = 0.016, and the estimated SD of effects across studies is τ = 0.13. Furthermore, Egger’s test of regression intercept revealed potential evidence of publication bias for FM (*t* = −2.8, *p*-value = 0.01). However, no adjustments to ES were made since there was no change in results when trim and fill technique [29] was performed. Funnel plot for FM is shown in Fig. 5A. According to the meta-regression, age did not explain to the variations in ES values for FM (−0.003 change in FM per 1-point age increase; 95% CI: −0.140 to 0.009; *p* = 0.645). There was no significant difference in effect between subgroup variables, including BMI, exercise type, study duration, and energy intake for FM (Table 3).

**Table 3:** Moderator analysis for body composition variables.

Body composition variable	Moderator variable	Moderator variable categories	*n*	ES	95% CI	Q_b_
FM (kg)	BMI	<25	7	−0.26^a^	−0.39 to −0.13	0.82
		≥25	6	−0.17^a^	−0.31 to −0.03	
	Exercise type	Aerobic	4	−0.29^a^	−0.44 to −0.13	2.56
		Resistance	6	−0.14^a^	−0.25 to −0.02	
		Concurrent	5	−0.22^a^	−0.36 to −0.09	
	Duration	≥8 weeks	8	−0.24^a^	−0.34 to −0.14	1.42
		<8 weeks	7	−0.16^a^	−0.25 to −0.06	
	Energy intake	Stable	7	−0.22^a^	−0.33 to −0.11	1.69
		TRE Hypocaloric	3	−0.27^a^	−0.45 to −0.1	
		Both Hypocaloric	5	−0.13^a^	−0.27 to −0.0004	
	Total	-	15	−0.20^a^	−0.28 to −0.12	57.8^a^
BF%	BMI	<25	6	−0.30^a^	−0.46 to −0.13	1.86
		≥25	2	−0.08	−0.35 to 0.19	
	Exercise type	Aerobic	3	−0.35^a^	−0.56 to −0.13	4.42
		Resistance	4	−0.1	−0.27 to 0.07	
		Concurrent	2	−0.35^a^	−0.61 to −0.1	
	Duration	≥8 weeks	4	−0.27^a^	−0.46 to −0.09	0.37
		<8 weeks	5	−0.20^a^	−0.36 to −0.03	
	Energy intake	Stable	4	−0.25^a^	−0.44 to −0.06	2.49
		TRE Hypocaloric	2	−0.39^a^	−0.66 to −0.11	
		Both Hypocaloric	3	−0.11	−0.32 to 0.10	
	Total	-	9	−0.23	−0.35 to −0.11	49.9^a^
FFM (kg)	BMI	<25	7	−0.08^a^	−0.15 to −0.01	2.8
		≥25	6	0.006	−0.07 to 0.08	
	Exercise type	Aerobic	4	−0.09^a^	−0.17 to 0.003	1.33
		Resistance	6	−0.03	−0.10 to 0.04	
		Concurrent	5	−0.02	−0.11 to 0.08	
	Duration	≥8 weeks	8	−0.05	−0.12 to 0.02	0.09
		<8 weeks	7	−0.04	−0.10 to 0.03	
	Energy intake	Stable	7	−0.04	−0.10 to 0.01	2.63
		TRE Hypocaloric	3	−0.12^a^	−0.24 to 0.001	
		Both Hypocaloric	5	−0.004	−0.08 to 0.08	
	Total	-	15	−0.04	−0.09 to 0.004	23.8^a^

Abbreviations: *FFM* Fat-free mass, *FM* Fat mass, *BF%* Body fat percent, *BMI* Body mass index, *Stable*, No difference in energy intake between groups pre to post; *TRE hypocaloric*, Intervention group has calorie deficit from pre to post; *both hypocaloric*, Both groups in calorie deficit from pre to post; *CI* Confidence interval, *ES* Effect size, *n* Number of studies, *Q*_*b*_ Cochran’s Q statistic.

^a^*p* < 0.10.

A small but significant mean reduction in BF% was observed after following TRE with exercise compared to the exercise-only control group (ES = −0.23, 95% CI = −0.35 to −0.11 and *p* < 0.001). Since the prediction interval is −0.64 to 0.18, the true ES can be as low as −0.64 and as high as 0.18. The overall ES may be equivalent to a 1.34% reduction in BF% which was determined by multiplying the ES for BF% by the pooled SD of BF% for the intervention group in the study with the largest number of subjects [39]. However, there was high heterogeneity between the studies for BF% as observed with Q-statistic (Q = 49.9, df = 8, and *p*-value < 0.001), I^2^ statistic (I^2^ = 83%), and Tau (τ^2^ = 0.03, and τ = 0.16). Egger’s regression test revealed potential evidence of publication bias for BF% (*t* = −5.4, *p*-value < 0.001). Since there was no change in results when trim and fill [29] technique was performed, no adjustments to ES was performed. The Funnel plot is shown in Fig. 5B. According to the meta-regression, age did not explain to the variations in ES values for BF% (0.004 change in BF% per 1-point age increase; 95% CI: −0.014 to 0.022; *p* = 0.638). The moderator analyses with BMI, exercise type, study duration, and energy intake did not explain the variation in ESs for BF% (Table 3).

There was no significant change in FFM in TRE with exercise compared to the control group who did exercise only (ES = −0.04, 95% CI = −0.09 to 0.004 and *p* = 0.07). The prediction interval is −0.17 to 0.09. In terms of FFM change in kg, there was a reduction of 0.06 kg of FFM when calculated as previously described [32]. Significant heterogeneity was also observed for FFM ESs between studies according to Q-statistic (Q = 23.8, df = 14, and *p*-value = 0.048, I^2^ statistic (I^2^ = 40.6%), and Tau (τ^2^ = 0.003 and τ = 0.05). Egger’s test of regression intercept result showed no evidence of publication bias for FFM (*t* = −0.46*, p* = 0.65) and the funnel plot also showed a symmetrical distribution (Fig. 5C). According to the meta-regression, age did not explain to the variations in ES values for FFM (−0.004 change in FFM per 1-point age increase; 95% CI: −0.010 to 0.003; *p* = 0.316). Any other study characteristics variables including BMI, exercise type, study duration, and energy intake did not result in a significant difference in effect on FFM according to the moderator analysis (*p* > 0.05) (Table 3).

## Discussion

While the impact of intermittent fasting with resistance training has been previously explored with a meta-analysis [18], the effects of TRE when combined with multiple types of exercise were unclear. Therefore, the present meta-analysis aimed to evaluate the effects of TRE with exercise on body composition outcomes compared to exercise-matched controls who did not follow TRE, analyzing studies with an intervention period of at least 4 weeks.

According to this meta-analysis, a small but statistically significant reduction in FM and BF% may occur following TRE with exercise ≥4 weeks without significant changes to FFM when compared to exercise-matched, unrestricted mealtime controls. When the ES for FM and BF% is converted into a clinically relevant measurement, the estimated additional loss of FM and BF% may be close to 1.3 kg and 1.3%, respectively. Nevertheless, prediction intervals suggest inconsequential impacts on FM and BF% in some populations, with potential variability in fat gain or loss. Interestingly, TRE with exercise did not cause a significant change in FFM compared to the control. Similar to the present findings, the meta-analysis conducted by Ashtary-Larky, et al. [18] reported a reduction in FM without significant changes in FFM when IF is combined with resistance training.

Several potential mechanisms may underlie the favorable effects of TRE with exercise, with unintentional energy restriction [11, 32, 35, 40, 43] being the main one. TRE may cause a spontaneous reduction in energy intake due to restricted eating window, which may result in a reduction in body weight [40, 44–46]. In the current analysis, the majority of the studies reported an energy deficit for the TRE group, either by design [34, 37, 39, 41] or unintentionally [32, 33, 35, 40]. However, the moderator analysis did not indicate a significant impact on body composition by the energy intake levels. Similar to calorie restriction, TRE stimulates AMP-activated protein kinase/Acetyl-CoA-Carboxylase (AMPK/ACC) signaling pathway [47]. This pathway is activated under conditions of low cellular energy availability, promoting ATP synthesis by enhancing fatty acid oxidation and upregulating glycolytic flux. However, the current meta-analysis is not able to provide any evidence for these mechanisms.

In the current analysis, all but one [40] of the 15 studies followed a TRE protocol with a feeding window during daylight hours [8, 9, 12, 31, 33, 34, 36–39, 41]. Thus, the feeding time for the intervention group is aligned with the light-dark cycle, whereas the CON groups typically ate well into the evening. In humans, the functions of nearly all organs and systems are regulated by circadian rhythms and exhibit daily oscillations [48]. Significant variations from this circadian rhythm, such as eating the majority of calories at night, have been shown to alter metabolism and can lead to obesity and other chronic metabolic diseases [49–51]. Proposed mechanisms of TRE are alignment of nutrient intake with the circadian rhythm [52], and hormonal changes due to TRE such as increased adiponectin, noradrenaline, growth hormone, and a decrease in cortisol [12, 36, 37, 53]. Similarly, calorie restriction has been shown to increase adiponectin and noradrenaline [54], but yet to be confirmed in humans. Since growth hormone is highly regulated by circadian rhythms, [55] alignment of feeding/fasting times with the light and dark cycle could be a mechanism for increased growth hormone observed in TRE. More research is needed, and studying the mechanisms is beyond the scope of this meta-analysis.

Moderator analyses based on BMI, type of exercise (aerobic, resistance, and concurrent), study duration, and energy intake did not find any significant impact on the variation in effect size for any of the main outcome variables. Heterogeneity observed in this meta-analysis suggests that variability in effect sizes is not fully explained by the study variables. While it would have been interesting to explore the impact of different timing of TRE protocols, studies on early TRE are limited. Thus, it was not possible to explore the effects of the timing of TRE in the moderator analysis at this time. Further investigation is necessary to fully understand the sources of heterogeneity in the observed effects.

The results of this study should be interpreted with caution due to certain limitations. Most studies included in the current meta-analysis are four weeks [8, 9, 36, 38, 39, 42] or eight weeks [12, 31, 34, 35, 37, 40, 41] in duration with only one [33] exceeding twelve weeks in length. Furthermore, only one study utilizing a resistance training protocol and TRE has exceeded a year [11]. This limitation prevents conclusions regarding any long-term body composition benefits of TRE against an unrestricted diet in an exercising population. Thus, longer-term studies are needed to determine the long-term effects of TRE with exercise on body composition. All of the studies in this analysis used self-reporting methods to document dietary intake, which often underreports energy intake [56]. Therefore, the true nature of energy intake in TRE participants is unknown, and further studies using more objective measures of energy intake are warranted. Since significantly high heterogeneity was observed for the body composition outcomes in the present meta-analysis, findings from this meta-analysis need to be interpreted carefully. Furthermore, dietary energy and protein intake may impact body composition changes in exercising individuals [57], but were not studied in the present meta-analysis. In addition, different body composition measuring devices were used in the studies that may have impacted outcomes. Of the 15 studies, five used bioelectrical impedance analysis (BIA) [31–33, 36, 37], one used skin fold testing [35], seven used dual-energy x-ray absorptiometry (DXA) [8, 9, 12, 34, 38, 40, 42], and two used a 4-compartment model [39, 41]. Although all these techniques have been validated, they do not always reflect similar changes in body composition. However, the random effects model and SMD’s we used in the meta-analysis accounts for the variability introduced by the different body composition methods (DXA, BIA, etc.). The generalizability of the findings is limited since most of the participants were metabolically healthy and experienced exercisers, with only a few studies on participants with BMI ≥ 30 kg/m^2^ [32, 34, 37].

## Conclusion

As per the findings of the current meta-analysis, TRE appears to induce a small decrease in FM and BF% while conserving FFM in adults adhering to a structured exercise regimen, as opposed to exercise-matched controls without temporal eating restrictions. Future investigations should focus on long-term studies utilizing various TRE protocols in diverse populations.

## Supplementary information

Supplemental Material
