# Supplementary material for: Effects of time-restricted eating with exercise on body composition in adults: a systematic review and meta-analysis
Source: Int J Obes (Lond). 2025 Jan 10;49(5):755–65. doi: 10.1038/s41366-024-01704-2 (PMC12095083; doi:10.1038/s41366-024-01704-2)
Supplement: Supplementary file 1 — Supplemental Material [file 41366_2024_1704_MOESM1_ESM.docx]

**Supplements**

**Meta-analysis search criteria**

**Research question**

**Does time-restricted eating with exercise improve body composition compared to exercise alone?**

Concept 1: Fasting [mesh] feeding behavior [mesh]

Keywords: time restricted eating, time restricted feeding, intermittent fasting, time restricted diet, time restricted meal, time restricted fasting, intermittent caloric restriction, intermittent energy restriction, intermittent feeding,

Concept 2: Exercise [mesh]

Aerobic exercise, anaerobic exercise, concurrent exercise, resistance exercise, exercise training, exercise performance, physical activity, circuit training, high intensity interval training, HIIT,

Concept 3: Body composition [mesh]

Body mass, fat mass, fat-free mass, lean mass, skeletal muscle mass,

**Search Strategy**

**Using PubMed**

Query 1: fasting[mesh] OR “feeding behavio*”[mesh] OR “time-restricted feeding”[tw] OR “time-restricted eating”[tw] OR “time-restricted diet”[tw] OR “time-restricted fasting”[tw] OR “intermittent fasting”[tw] OR “intermittent feeding”[tw] OR “intermittent caloric restriction”[tw] OR “intermittent energy restriction”[tw] (8032 results)

Query 2: Exercise[mesh] OR “exercise training”[tw] or “physical activity”[tw] OR “resistance training”[tw] OR “anaerobic training”[tw] OR “aerobic training”[tw] OR “concurrent training”[tw] OR “circuit training”[tw] OR “high intensity interval training”[tw] OR HIIT[tw] (27165 results)

Query 3: “body composition” [mesh] OR “fat mass”[tw] OR “fat-free mass”[tw] OR “lean mass”[tw] OR “skeletal muscle mass”[tw] (5751 results)

Query 4: #1 AND #2 AND #3 (109 results)

(fasting [mesh] OR “time-restricted feeding” OR “time-restricted eating” OR “time-restricted diet” OR “time-restricted meal” OR “time-restricted fasting” OR “intermittent fasting” OR “intermittent feeding” OR “intermittent caloric restriction” AND (“Exercise”[mesh] OR “training” OR “exercise training” or “physical activity” OR “resistance training” OR “anaerobic training” OR “aerobic training” OR “concurrent training” OR “circuit training” OR “high intensity interval training”) AND (“body composition” [mesh] OR “fat mass” OR “fat-free mass” OR “lean mass” OR “skeletal muscle mass”

**Using EBSCOhost**

Databases: Academic search premier, CINAHL, MEDLINE, SPORTDiscus

***Search terms:***

“time-restricted feeding” OR “time-restricted eating” OR “time-restricted diet” OR “time-restricted fasting” OR “intermittent fasting” OR “intermittent feeding” OR “intermittent caloric restriction” OR “intermittent energy restriction”

AND

“exercise training” OR "aerobic exercise" OR "resistance exercise" OR "anaerobic exercise" OR “physical activity” OR “resistance training” OR “anaerobic training” OR “aerobic training” OR “concurrent training” OR “circuit training” OR “high intensity interval training” (426 articles but 150 excluded automatically due to duplicates. Exported to endnote: 276)

**Using SCOPUS**

TITLE-ABS-KEY ( {time restricted feeding}  OR  {time restricted eating}  OR  {intermittent fasting} OR {time restricted diet} OR {time restricted fasting} OR {intermittent feeding} OR {intermittent caloric restriction} OR {intermittent energy restriction}  AND  {exercise}  OR  {aerobic exercise}  OR  {aerobic training}  OR  {anaerobic exercise}  OR  {anaerobic training}  OR  {resistance training}  OR  {high intensity interval training} OR {HIIT} OR {physical activity} OR {circuit training} )  (469 articles)
